# Urethral Calculus in a Bay Gelding

**Published:** 1881-10

**Authors:** John Cuff

**Affiliations:** 150 Jane St., New York City


### IX.—URETHRAL CALCULUS IN A BAY GELDING.

REPORTED BY JOHN CUFF, D.V.S.

On July 13th, 1881, I was summoned to see a bay gelding, aged 8 years, the property of the Consumers' Ice Co., of this city.

The animal in question I found to be suffering severe colicky pains, and occasionally stretching himself, evidently with a desire to micturate. I ordered the following draught:

℞

Tr. Opii, et.

Sprts. nitros, dulc. et.

Ether Sulph. āā., ℥ i.

Olei Lini, ℥ vij.

Misce; sig.; at a draught.

As no improvement followed after a few hours, I made a rectal examination, and found the bladder distended, and diagnosticated retention of urine.

When an attempt was made to introduce a catheter into the bladder, it was found that the urethra was plugged by a hard, resisting substance, and the retention of urine found to be secondary to a urethral calculus.

To allow the obstruction to remain would have soon terminated the life of the animal. I again ordered

℞
Tr. Opii, ℥ i.
Oleum Lini, ℥ vij.

Misce; sig.; at once, and decided upon an early operation.

July 14th, at 8 o'clock A.M., I found the animal still suffering intense pain, and unable to micturate. I had him immediately transferred to my infirmary, 150 Jane street. When the horse arrived, the breathing was hurried and shallow; pulse, 70 per minute; temperature, 108° F.

Everything being ready, at 1 o'clock. P.M. the animal was cast. Five cuts were made in the integuments, and the incision extended through the underlying structures and urethra until the calculus was exposed and removed. The calculus was egg-shaped and weighed two ounces. The wound was closed with a few interrupted sutures.

Time of operation, including casting, was only seven minutes.

The gelding, as soon as loosed, rose to his feet and voided, quite freely, a large quantity of urine. The symptoms of pain and uneasiness rapidly disappeared.

The after treatment was:

℞

Spts. Frumenti, ℥ ij.

Decot. Lini, Oj.

Misce; sig.; draught, and a diet of bran mash, oatmeal gruel and demulcent drinks.

Two hours after the operation the breathing was still hurried; pulse, 48; temperature, 102° F. Ordered:

℞

Spts. Frumente, ℥ iv.
Tr. Opii, ℥ i.
Oleum Lini, ℥ vij.

Mix; sig.; drench.

At 6 o'clock P.M. the same day the breathing had become nearly normal; pulse, 40; temperature, 100° F. Ordered:

℞

Hydrastis Canadenss, ℥ i.

Inf. Lini, ℥ vij.

Misce; sig.; at one draught.

July 15th, 6 o'clock A.M., patient doing remarkably well; all evidence of pain had disappeared; a small part of the urine passed through the artificial opening. The horse was allowed a liberal diet, and all medicine stopped except the alcoholic stimulants, which were continued in small quantities until July 30th. At this date the artificial opening was found to be thoroughly united, and the sutures were removed. A cathartic was given.

August 19th, the patient was discharged cured, and seven days later was at work, and has been ever since, apparently as well at before the attack.

150 Jane St., New York City.

